# Supplementary material for: Profiling the plasmid conjugation potential of urinary Escherichia coli
Source: Microb Genom. 2022 May 10;8(5):mgen000814. doi: 10.1099/mgen.0.000814 (PMC9465074; doi:10.1099/mgen.0.000814)
Supplement: Supplementary material 1 [file mgen-8-814-s001.pdf]

Profiling the plasmid conjugation potential of urinary *E. coli*

Cesar Montelongo Hernandez, Catherine Putonti, Alan J. Wolfe

Supplemental Information

Supplemental Table 1. List of urinary *E. coli* isolates used in this study

Strain	Taxonomy	Assembly	Level	WGS	BioSample
UMB0103	*Escherichia coli*	GCA_003892645.1	Contig	RRWT00000000	SAMN09665164
UMB0149	*Escherichia coli*	GCA_003892555.1	Contig	RRWS00000000	SAMN09665165
UMB0276	*Escherichia coli*	GCA_003892545.1	Contig	RRWR00000000	SAMN09665166
UMB0527	*Escherichia coli*	GCA_003892535.1	Contig	RRWQ00000000	SAMN09665167
UMB0731	*Escherichia coli*	GCA_003892485.1	Contig	RRWP00000000	SAMN09665168
UMB0906	*Escherichia coli*	GCA_003886695.1	Contig	RRWO00000000	SAMN09665169
UMB0923	*Escherichia coli*	GCA_003892635.1	Contig	RRWN00000000	SAMN09665170
UMB0928	*Escherichia coli*	GCA_003892445.1	Contig	RRWM00000000	SAMN09665171
UMB0931	*Escherichia coli*	GCA_003886495.1	Contig	RRWL00000000	SAMN09665172
UMB0933	*Escherichia coli*	GCA_003886675.1	Contig	RRWK00000000	SAMN09665173
UMB0934	*Escherichia coli*	GCA_003892475.1	Contig	RRWJ00000000	SAMN09665174
UMB0939	*Escherichia coli*	GCA_003885295.1	Contig	RRUR00000000	SAMN09665218
UMB0949	*Escherichia coli*	GCA_003892435.1	Contig	RRWI00000000	SAMN09665175
UMB1012	*Escherichia coli*	GCA_003886455.1	Contig	RRWH00000000	SAMN09665176
UMB1091	*Escherichia coli*	GCA_003886445.1	Contig	RRWG00000000	SAMN09665177
UMB1093	*Escherichia coli*	GCA_003885215.1	Contig	RRUQ00000000	SAMN09665219
UMB1160	*Escherichia coli*	GCA_003892605.1	Contig	RRWF00000000	SAMN09665178
UMB1162	*Escherichia coli*	GCA_003892455.1	Contig	RRWE00000000	SAMN09665179
UMB1180	*Escherichia coli*	GCA_008726795.1	Contig	VYWI00000000	SAMN12797014
UMB1193	*Escherichia coli*	GCA_003892595.1	Contig	RRWC00000000	SAMN09665181
UMB1195	*Escherichia coli*	GCA_008726745.1	Contig	VYWF00000000	SAMN12797017
UMB1202	*Escherichia coli*	GCA_003886395.1	Contig	RRWA00000000	SAMN09665183
UMB1220	*Escherichia coli*	GCA_003886385.1	Contig	RRVZ00000000	SAMN09665184
UMB1221	*Escherichia coli*	GCA_003885055.1	Scaffold	RRUG00000000	SAMN10411422
UMB1223	*Escherichia coli*	GCA_003886375.1	Contig	RRVY00000000	SAMN09665185
UMB1225	*Escherichia coli*	GCA_008726695.1	Contig	VYWD00000000	SAMN12797019

UMB1228	*Escherichia coli*	GCA_003886655.1	Contig	RRVW00000000	SAMN09665187
UMB1229	*Escherichia coli*	GCA_003886345.1	Contig	RRVV00000000	SAMN09665188
UMB1284	*Escherichia coli*	GCA_003892355.1	Contig	RRVU00000000	SAMN09665189
UMB1285	*Escherichia coli*	GCA_003886635.1	Contig	RRVT00000000	SAMN09665190
UMB1335	*Escherichia coli*	GCA_003886615.1	Contig	RRVS00000000	SAMN09665191
UMB1337	*Escherichia coli*	GCA_003886325.1	Contig	RRVR00000000	SAMN09665192
UMB1346	*Escherichia coli*	GCA_003886295.1	Contig	RRVQ00000000	SAMN09665193
UMB1347	*Escherichia coli*	GCA_003886285.1	Contig	RRVP00000000	SAMN09665194
UMB1348	*Escherichia coli*	GCA_003886275.1	Contig	RRVO00000000	SAMN09665195
UMB1354	*Escherichia coli*	GCA_003886225.1	Contig	RRVN00000000	SAMN09665196
UMB1356	*Escherichia coli*	GCA_003886245.1	Contig	RRVM00000000	SAMN09665197
UMB1358	*Escherichia coli*	GCA_003886195.1	Contig	RRVL00000000	SAMN09665198
UMB1359	*Escherichia coli*	GCA_003886185.1	Contig	RRVK00000000	SAMN09665199
UMB1360	*Escherichia coli*	GCA_003886565.1	Contig	RRVJ00000000	SAMN09665200
UMB1362	*Escherichia coli*	GCA_003886175.1	Contig	RRVI00000000	SAMN09665201
UMB1526	*Escherichia coli*	GCA_003886105.1	Contig	RRVH00000000	SAMN09665202
UMB1727	*Escherichia coli*	GCA_003886135.1	Contig	RRVG00000000	SAMN09665203
UMB2019	*Escherichia coli*	GCA_003886115.1	Contig	RRVF00000000	SAMN09665204
UMB2055	*Escherichia coli*	GCA_003886095.1	Contig	RRVE00000000	SAMN09665205
UMB2321	*Escherichia coli*	GCA_003886555.1	Contig	RRVD00000000	SAMN09665206
UMB2328	*Escherichia coli*	GCA_003886545.1	Contig	RRVC00000000	SAMN09665207
UMB3538	*Escherichia coli*	GCA_003886535.1	Contig	RRVB00000000	SAMN09665208
UMB3641	*Escherichia coli*	GCA_003885305.1	Contig	RRUO00000000	SAMN09665221
UMB3643	*Escherichia coli*	GCA_003885095.1	Scaffold	RRUF00000000	SAMN10411421
UMB4656	*Escherichia coli*	GCA_003886515.1	Contig	RRVA00000000	SAMN09665209
UMB4716	*Escherichia coli*	GCA_003885995.1	Contig	RRUN00000000	SAMN09665222
UMB4746	*Escherichia coli*	GCA_003886045.1	Contig	RRUZ00000000	SAMN09665210
UMB5337	*Escherichia coli*	GCA_003886035.1	Contig	RRUY00000000	SAMN09665211
UMB5814	*Escherichia coli*	GCA_003886015.1	Contig	RRUX00000000	SAMN09665212
UMB5924	*Escherichia coli*	GCA_003886005.1	Contig	RRUW00000000	SAMN09665213

UMB5978	*Escherichia coli*	GCA_003885915.1	Contig	RRUV00000000	SAMN09665214
UMB6454	*Escherichia coli*	GCA_003885245.1	Contig	RRUU00000000	SAMN09665215
UMB6611	*Escherichia coli*	GCA_003885875.1	Contig	RRUT00000000	SAMN09665216
UMB6653	*Escherichia coli*	GCA_003885965.1	Scaffold	RRUS00000000	SAMN09665217
UMB6655	*Escherichia coli*	GCA_003885255.1	Contig	RRUL00000000	SAMN09665225
UMB6713	*Escherichia coli*	GCA_003885145.1	Contig	RRUK00000000	SAMN09665226
UMB6721	*Escherichia coli*	GCA_003885125.1	Scaffold	RRUJ00000000	SAMN09665227
UMB6890	*Escherichia coli*	GCA_003885035.1	Contig	RRUI00000000	SAMN09665228
UMB7431	*Escherichia coli*	GCA_003885225.1	Scaffold	RRUH00000000	SAMN09665229

Supplemental Table 3. Transfer gene function and repository access

***tra* gene**	**UniProtKB**
Transcription regulation	
finO	P22707
traJ	P06626
traR	P41065
ProPilin maturation	
traA	P04737
traQ	P18033
traX	P22709
T4SS core proteins	
traB	P41067
traK	P41066
traV	P41069
Pilus assembly/extension	
traF	P14497
trbB	P18035
traC	P18004
traW	P18472
trbC	P18473
traP	P41068
traE	P08322
traL	P08321
Pilus retraction	
traH	P15069
trbI	P18006
Mating pair stabilization	
traN	P24082
traG	P33790
traU	P18471
Relaxase	
traI	P14565
Relaxosome accessory	
traY	P06627
traM	P10026
traD	P09130
Surface/entry exclusion	
traT	P13979
traS	P09129

Supplemental Table 4. VirB-VirD4 and transfer gene homologues profiled in urinary *E. coli*

Conjugation gene	tra homologues	UniProtKB
virB1		A0A0R6LN99
virB2	*traA*	A0A4C4D6J9
virB3	*traL*	A0A4C4D6J9
virB4	*traC*	A0A0A1DZA0
virB5	*traE*	G8GYF8
virB6	*traG*	G1CCR5
virB7	*traV*	B0ZDY1
virB8	*traG*	G8GYF5
virB9	*traK*	A0A0C5F896
virB10	*traB*	A0A0R6LEV8
virB11		A0A0C5EWI7
virD2	*traI*	A0A482M9K1
virD4	*traD*	A0A381CEJ0
ptleE		A0A377MWU4
trbC	*traA*	A0A1P8KHV0
trbE	*traC*	Q05807
trbI	*traB*	P18006
trbP	*traX*	W6AWJ8
trwB	*traD*	Q04230
trwC	*traI*	Q47673
trwM		O50329

Supplemental Table 5. Growth of urinary *E. coli* isolates on antibiotic plates

			LB	Amp	Cm	Kan	Spec	Tet
		**Total**	**68**	**28**	**1**	**3**	**8**	**16**
**Strain**	**Inc group**	**# abx that it grew on**	**100**	**41.1765**	**1.47059**	**4.41176**	**11.7647**	**23.5294**
B	Control	0	Yes	No	No	No	No	No
C	Control	0	Yes	No	No	No	No	No
K-12	Control	0	Yes	No	No	No	No	No
UMB0103	IncF	3	Yes	Yes	Yes	No	No	Yes
UMB0149	Inc-various	0	Yes	No	No	No	No	No
UMB0276	No inc	1	Yes	Yes	No	No	No	No
UMB0527	IncF	0	Yes	No	No	No	No	No
UMB0731	Col	0	Yes	No	No	No	No	No
UMB0906	IncF	1	Yes	Yes	No	No	No	No
UMB0923	Inc-various	0	Yes	No	No	No	No	No
UMB0928	IncF	2	Yes	Yes	No	No	No	Yes
UMB0931	IncF	2	Yes	Yes	No	No	No	Yes
UMB0933	IncF	1	Yes	No	No	No	Yes	No
UMB0934	IncF	2	Yes	Yes	No	No	No	Yes
UMB0939	Col	2	Yes	Yes	No	No	No	Yes
UMB0949	IncF	2	Yes	Yes	No	No	No	Yes
UMB1012	IncF	1	Yes	Yes	No	No	No	No
UMB1091	IncF	3	Yes	Yes	No	No	Yes	Yes
UMB1093	IncF	1	Yes	Yes	No	No	No	No
UMB1160	IncF	1	Yes	No	No	No	Yes	No
UMB1162	IncF	1	Yes	No	No	No	No	Yes
UMB1180	Col	0	Yes	No	No	No	No	No
UMB1193	IncF	3	Yes	Yes	No	No	Yes	Yes
UMB1195	IncF	0	Yes	No	No	No	No	No
UMB1202	IncF	0	Yes	No	No	No	No	No
UMB1220	No inc	0	Yes	No	No	No	No	No
UMB1221	IncF	1	Yes	No	No	No	No	Yes
UMB1223	IncF	2	Yes	Yes	No	No	No	Yes
UMB1225	No inc	0	Yes	No	No	No	No	No
UMB1228	Inc-various	0	Yes	No	No	No	No	No
UMB1229	IncF	3	Yes	Yes	No	No	Yes	Yes
UMB1284	IncF	4	Yes	Yes	No	Yes	Yes	Yes
UMB1285	IncF	0	Yes	No	No	No	No	No

UMB1335	IncF	1	Yes	Yes	No	No	No	No
UMB1337	IncF	1	Yes	Yes	No	No	No	No
UMB1346	IncF	0	Yes	No	No	No	No	No
UMB1347	IncF	0	Yes	No	No	No	No	No
UMB1348	IncF	1	Yes	Yes	No	No	No	No
UMB1354	No inc	0	Yes	No	No	No	No	No
UMB1356	No inc	0	Yes	No	No	No	No	No
UMB1358	No inc	0	Yes	No	No	No	No	No
UMB1359	No inc	0	Yes	No	No	No	No	No
UMB1360	IncF	1	Yes	Yes	No	No	No	No
UMB1362	IncFI	2	Yes	Yes	No	No	No	Yes
UMB1526	No inc	1	Yes	Yes	No	No	No	No
UMB1727	IncFII	1	Yes	Yes	No	No	No	No
UMB2019	Col	0	Yes	No	No	No	No	No
UMB2055	Inc-various	0	Yes	No	No	No	No	No
UMB2321	Inc-various	0	Yes	No	No	No	No	No
UMB2328	Inc-various	0	Yes	No	No	No	No	No
UMB3538	IncF	2	Yes	Yes	No	Yes	No	No
UMB3641	IncF	2	Yes	Yes	No	No	Yes	No
UMB3643	Inc-various	1	Yes	Yes	No	No	No	No
UMB4656	IncF	1	Yes	Yes	No	No	No	No
UMB4716	IncF	0	Yes	No	No	No	No	No
UMB4746	IncF	0	Yes	No	No	No	No	No
UMB5337	No inc	0	Yes	No	No	No	No	No
UMB5814	IncF	0	Yes	No	No	No	No	No
UMB5924	No inc	4	Yes	Yes	No	Yes	Yes	Yes
UMB5978	IncF	0	Yes	No	No	No	No	No
UMB6454	IncF	0	Yes	No	No	No	No	No
UMB6471	IncF	0	Yes	No	No	No	No	No
UMB6611	IncF	0	Yes	No	No	No	No	No
UMB6655	IncF	0	Yes	No	No	No	No	No
UMB6713	IncF	1	Yes	Yes	No	No	No	No
UMB6721	IncF	1	Yes	No	No	No	No	Yes
UMB6890	IncF	0	Yes	No	No	No	No	No
UMB7431	IncF	0	Yes	No	No	No	No	No

Supplemental Table 6. Conjugation of urinary *E. coli* plasmids

Isolate	Role in conjugation	Urinary plasmid replicon	Conjugation genes profiled prior to conjugation	Tc resistance predicted[1]	Growth on Tc plate	Growth on Cm plate	Growth on Tc Cm plate	Produces transconjugants on Tc Cm plates[2]
K-12 MG1655	Plasmid recipient	None	None	No	No	Yes	No	Yes
UMB1223	Plasmid donor	IncFA, IncFIB(AP001918), IncFII(pRSB107)	*finO, traA, traR, traX, traB, traK, traV, traF, trbB, traC, traW, trbC, traP, traE, traL, traH, trbI, traG, traU, traI, traM, traD, traT*	Yes	Yes	No	No	Yes
UMB1284	Plasmid donor	IncFIA, IncFII, IncX4	*traG, traT, virB2, virB3, virB4, virB5, virB8*	Yes	Yes	No	No	Yes
UMB0939	Plasmid donor without conjugation system (negative control)	Col(MG828), ColRNAI	None	No	Yes	No	No	No

[1] Contig with gene for Tc resistance has homology to plasmid

[2] Urinary *E. coli* conjugated with *E. coli* K-12 strain MG1655 with chloramphenicol resistance selection marker

Supplemental Table 7. Conjugation genes identified in pU1223

Annotation ID	Gene	Query Cover	E value	Per. Ident	Acc. Len	Accession
GJKJIFEI_00008	*finO*	100%	8.00E-132	100.00%	188	KUT64489.1
GJKJIFEI_00009	*traX*	100%	2.00E-177	99.60%	248	WP_053879992.1
GJKJIFEI_00010	*traI*	100%	0	99.94%	1756	WP_172694226.1
GJKJIFEI_00011	*traD*	100%	0	99.73%	735	EHI0361888.1
GJKJIFEI_00012	*traY*	100%	0	100.00%	287	PHN11796.1
GJKJIFEI_00013	*traT*	100%	0	100.00%	293	ANK07087.1
GJKJIFEI_00014	*TraS_2*	100%	6.00E-117	99.39%	163	CTS32472.1
GJKJIFEI_00015	*traG*	100%	0	100.00%	941	HAM6569033.1
GJKJIFEI_00016	*traH*	100%	0	99.78%	457	WP_061089898.1
GJKJIFEI_00017	*trbF_2*	100%	2.00E-92	100.00%	141	WP_001348758.1
GJKJIFEI_00018	*trbJ_2*	100%	6.00E-61	98.95%	95	KUV34015.1
GJKJIFEI_00019	*trbB*	100%	2.00E-130	99.45%	181	WP_087507405.1
GJKJIFEI_00020	*traQ*	100%	7.00E-61	98.94%	94	HAJ2583838.1
GJKJIFEI_00021	*trbA_2*	100%	1.00E-68	100.00%	127	ESA77523.1
GJKJIFEI_00022	Transposase	100%	3.00E-120	99.40%	167	AVJ76634.1
GJKJIFEI_00023	Transposase	100%	0	99.69%	326	HAW2113186.1
GJKJIFEI_00024	*traF*	100%	0	100.00%	257	ARX27828.1
GJKJIFEI_00025	*trbE*	100%	9.00E-54	100.00%	89	WP_033554899.1
GJKJIFEI_00026	*traN*	100%	0	99.84%	616	HAJ2996344.1
GJKJIFEI_00027	*trbC*	100%	9.00E-156	100.00%	216	ARX27831.1
GJKJIFEI_00028	*HP_1*	100%	1.00E-67	100.00%	109	WP_139581483.1
GJKJIFEI_00029	*traU*	100%	0	99.70%	330	WP_021528459.1
GJKJIFEI_00030	*traW*	100%	2.00E-151	100.00%	228	RBW13522.1
GJKJIFEI_00031	*trbI*	100%	2.00E-88	99.22%	133	ADL14037.1
GJKJIFEI_00032	*traC*	100%	0	99.89%	875	WP_157352444.1
GJKJIFEI_00033	*HP_2*	100%	2.00E-15	100.00%	48	EHV46867.1
GJKJIFEI_00034	*traV*	100%	4.00E-120	99.42%	171	WP_097762797.1
GJKJIFEI_00035	*trbD_2*	100%	2.00E-80	100.00%	127	HAL3406214.1
GJKJIFEI_00036	*traP*	100%	5.00E-138	100.00%	188	WP_001617877.1
GJKJIFEI_00037	*traB*	100%	0	100.00%	532	WP_077758417.1

GJKJIFEI_00038	*traK*	100%	4.00E-175	99.59%	242	WP_050008704.1
GJKJIFEI_00039	*traE*	100%	4.00E-136	99.47%	188	WP_032153574.1
GJKJIFEI_00040	*traL*	100%	4.00E-70	100.00%	109	WP_160372682.1
GJKJIFEI_00041	*traA*	100%	4.00E-77	99.17%	120	WP_069067339.1
GJKJIFEI_00042	*yraY_2*	100%	2.00E-45	100.00%	81	WP_049086340.1
GJKJIFEI_00043	*traJ_2*	100%	7.00E-49	100.00%	83	TFM56752.1
GJKJIFEI_00044	*HP_3*	97%	9.00E-19	97.67%	43	EGR70770.1
GJKJIFEI_00045	*HP_4*	100%	1.00E-32	100.00%	59	AKE87743.1
GJKJIFEI_00046	*traM*	100%	2.00E-86	99.21%	127	HAX8410798.1

Supplemental Table 8. Conjugation genes identified in pU1284

Annotation ID	Gene	Query Cover	E value	Per. Ident	Acc. Len	Accession
OJDLIIBG_00019	*T4SS_1*	100%	1.00E-65	99.01%	101	EFH9106740.1
OJDLIIBG_00020	*T4SS_2*	100%	3.00E-98	99.28%	140	OJM06430.1
OJDLIIBG_00021	*virD4_2*	100%	0	99.84%	620	WP_187440460.1
OJDLIIBG_00022	*virB11_2*	100%	0	99.71%	342	WP_089644540.1
OJDLIIBG_00023	*virB10_2*	100%	0	99.73%	372	EES3164312.1
OJDLIIBG_00024	*virB9*	100%	0	99.67%	302	WP_137486256.1
OJDLIIBG_00025	*virB8*	100%	5.00E-166	99.56%	228	WP_096190789.1
OJDLIIBG_00026	*hypo_vir*	100%	5.00E-35	100.00%	67	WP_000903502.1
OJDLIIBG_00027	*virB6_2*	55%	2.00E-10	84.85%	397	AQZ20251.1
OJDLIIBG_00028	*T4SS_3*	100%	0	100.00%	364	WP_125121539.1
OJDLIIBG_00029	*virB5*	100%	6.00E-174	99.58%	238	EFD0911387.1
OJDLIIBG_00030	*virB4*	100%	0	99.78%	915	EFB7338537.1
OJDLIIBG_00031	*virB2_2*	100%	1.00E-69	100.00%	127	ARH02006.1
OJDLIIBG_00032	*virB1_2*	100%	4.00E-150	99.51%	206	WP_032239609.1
OJDLIIBG_00138	*traD_2*	99%	1.00E-73	100.00%	119	EER1412495.1
OJDLIIBG_00139	*HP_5*	100%	0	99.59%	245	EFF9468201.1
OJDLIIBG_00140	*traT*	100%	1.00E-175	100.00%	244	AET14958.1
OJDLIIBG_00141	*traS_3*	100%	2.00E-112	98.11%	159	SRA12240.1

OJDLIIBG_00142	*traG*	100%	0	99.89%	940	WP_097760672.1
OJDLIIBG_00143	*traH*	100%	0	100.00%	332	GDF19761.1
